# A comparison of emotional wellbeing and burnout of primary care professionals in 2014 and 2021

**DOI:** 10.3389/fpubh.2022.1062437

**Published:** 2023-01-11

**Authors:** Alejandro Abad, Araceli Fuentes, Eugeni Paredes, Sofia Godoy, Sara Perera, Oriol Yuguero

**Affiliations:** ^1^Faculty of Medicine, Universitat de Lleida, Lleida, Spain; ^2^Primary Health Division, Catalan Health Institute, Lleida, Spain; ^3^ERLab, IRBLLEIDA, Lleida, Spain

**Keywords:** empathy, burnout, SARS-CoV-2, emotional exhaustion, personal accomplishment

## Abstract

**Background:**

Due to the pandemic that started in February–March 2020 and after many years of economic restrictions suffered by our health system, the levels of stress, exhaustion and suffering among health workers has increased.

**Objective:**

Our study aims to perform a comparative analysis of the degree of burnout and emotional wellbeing among health professionals between 2014 and 2021.

**Methods:**

This is a comparative descriptive study of two cohorts of primary care professionals of the Lleida health region (SPAIN). We have one cohort from 2014 and another from 2021 with the same selection criteria. Burnout was assessed using the Maslach Burnout Inventory (MBI-HSS) test. Gender, age, professional category and work environment were also evaluated.

**Results:**

We obtained a response rate in 2014 of 52.7% (*n* = 267) and of 41.4% (*n* = 217) in 2021 with similar sociodemographic characteristics. There are significant differences (*p* < 0.001) in the three categories of burnout. The high scores for emotional exhaustion and depersonalization have increased, rising between 2014 and 2021 from 23.2 to 60.8% and from 12.4 to 42.4%, respectively. However, there is also a significant increase in high personal accomplishment, rising from 9.0% in 2014 to 26.7%. We have also detected differences depending on age and professional role.

**Conclusion:**

This study shows worsening burnout levels of primary care professionals in our region, specifically emotional exhaustion and depersonalization. However, it also shows that during the pandemic, personal accomplishment was reinforced.

## Introduction

Burnout syndrome is currently considered by the WHO as an occupational disease. It was first described by the psychoanalyst Herbert J. Freudenberger in 1973 as a set of non-specific medical-biological and social symptoms. In the recent revision of the International Classification of Diseases (ICD-11) (1), burnout is described as a syndrome conceptualized as resulting from chronic workplace stress that has not been successfully managed. It is a three-dimensional syndrome defined by high emotional exhaustion, high depersonalization and increased mental distance from one's job, and reduced personal accomplishment and professional efficacy (2, 3).

Studies conducted during 2015 in the field of primary care, especially in our region, show that one-third of health professionals were suffering burnout (4, 5) and that it affected young people and women to a greater extent (6). Probably due to more implication in professional relationship and more emotional exhaustion.

With the arrival of the coronavirus pandemic in February–March 2020 and the situation of extreme work overload that this brought about, in a health system already subject to highly deficient conditions both due to a lack of personnel and material resources, including a shortage of personal protective equipment, the level of stress, exhaustion and suffering that was caused among health workers was enormous. Indeed, it was intuited that burnout must have worsened greatly, with the consequent repercussions on the quality of our health system. Studies in other regions have already demonstrated this (7–9) and results from China (10), the first country affected by the pandemic, have revealed some strategies to reduce burnout. However, it remains to be explored whether only emotional state is affected or if the pandemic has caused changes in other spheres of burnout.

Our study aims to analyse the degree of burnout and emotional wellbeing among health professionals (physicians and nurses) in our region during the coronavirus pandemic and compare it with that detected in 2014 in the same area, assessing the differences in the three categories of burnout, taking into account age groups, gender, length of service, and rural or urban setting.

## Methods

### Study design

This is a comparative descriptive study of two samples of primary care professionals (family doctors and nursing staff) from the Lleida health region.

### Setting

The Lleida Health Region has a target population of ~400,000 people, with 22 health centers throughout the territory and two public emergency care hospitals.

### Participants

We have one cohort from 2014 and another from 2021 consisting of both family doctors and nurses.

### Variables

#### Burnout level of professionals

Burnout was measured using the Spanish version of the MBI-HSS (MP) (Maslach Burnout Inventory for Medical Personnel) which consists of 22 items that assess feelings of emotional burnout, depersonalization and (decreased) personal accomplishment, with three scales Emotional Exhaustion, Depersonalization, and Personal Accomplishment, respectively.

Each item is scored on a 7-point Likert frequency scale ranging from “never” (0) to “every day” (6). The Spanish version was validated by Moreno-Jimenez et al. (11) and used previously by our research group (12, 13). Physicians and nurses were divided into three groups (low, medium and high burnout) according to scores for each of the three subscales of the MBI. The cut-off points used for the three categories were based on a previous project (14) and are described in other articles by the group (15). For Emotional Exhaustion low <19, medium 19–26, high >26; Depersonalization: low <6, medium 6–9, high >9; Personal Achievement: low >39, medium 34–39, high <34.

The MBI is widely recognized and has been administered to physicians and nurses both inside and outside Spain in numerous studies (16–18). The reliability of the instrument was tested by calculating Cronbach's alpha, which was 0.733 for the MBI. The three subscales showed good internal reliability with Cronbach's alpha coefficients above 0.7. Exploratory factor analysis revealed five factors with eigenvalues greater than 1. Factor analysis showed a relatively satisfactory fit of the three-factor structure (χ^2^/df = 2.6, SRMR = 0.07, RMSEA = 0.08, TLI = 0.87, CFI = 0.89).

### Other explanatory variables

We used other sociodemographic variables we deemed might be confounders of and could be related with the degree of burnout. We evaluated gender, age (31–40; 41–50; >50), professional category (physician/nurse), the work environment (urban/rural). Finally, the years of the surveys (2014/2021) were evaluated.

### Study size

To achieve the final sample we established the different inclusion and exclusion criteria.

### Inclusion criteria in both 2014 and 2021

Primary care professionals (doctors and nurses) who work regularly in their health center either in rural or urban areas. In 2014, there were 507 professionals in the region who met that criterion, and in 2021 there were 525 professionals.

### Exclusion criteria

Failure to sign the informed consent or having expressed the will not to participate in the project.

All primary care professionals who met the requirements were contacted *via* email and provided with the survey *via* email. In the first period, this took place between May and July 2014, and between January and February 2021 for the second cohort.

### Statistical methods

Our main analysis was the evaluation of the differences between the classification of MBI HSS (MP) subscales in two samples of health professionals collected in 2014 and 2021 The samples were compared using the χ^2^ test for their MBI subscales classification results as well as sample distribution by age group, sex, professional category and work environment.

An ordinal logistic regression model was fitted for the levels of each of the three areas of burnout (emotional exhaustion, depersonalization, and personal accomplishment) to adjust the differences between the 2 years for the characteristics of the health professionals and identify which of them had a modifier effect on the progression of the values of these variables (i.e., significant interactions with year). Possible interactions between health professionals' sex or age group variables were also tested and considered in the model whenever significant according to the likelihood ratio test.

Data were described by absolute and relative frequencies. Statistical analysis was performed with R (19).

### Ethics

The study was approved in both years by the Research Ethics Committee of the IDIAP Jordi Gol, and the confidentiality of the participants' data was respected. All participants gave their consent to take part in the study. The investigation was carried out following the protocols of the Declaration of Helsinki and in accordance with Spanish Organic Law 3/2018, on the Protection of Personal Data, and Regulation 2016/670 of the European Parliament.

## Results

In our samples, levels of burnout of primary care professionals had worsened significantly in 2021 compared to 2014. The response rate in 2014 was 52.7% (*n* = 267) and 41.4% (*n* = 217) in 2021.

A description of the two study samples can be seen in Table 1. There are no significant differences in age groups between the two samples. In both cohorts, the most predominant group contains professionals aged over 50 years and women professionals. There are no significant differences with respect to gender or professional category, which facilitates sample comparison. However, in 2021, there was greater participation of professionals from the urban than from the rural sphere (*p* < 0.005).

**Table 1:** Samples description.

**Burnout 2021 vs. 2014 in PC**				
	**2014**	**2021**	***p*.overall**	***N***
**Comparison**				
**Age (years)**				
31–40	60 (22.5%)	35 (16.1%)	0.202	484
41–50	95 (35.6%)	87 (40.1%)		
>50	112 (41.9%)	95 (43.8%)		
**Gender**				
Men	58 (21.7%)	42 (19.4%)	0.616	483
Women	209 (78.3%)	174 (80.6%)		
**Work environment**				
Urban	111 (41.6%)	198 (91.2%)	<0.001	484
Rural	156 (58.4%)	19 (8.76%)		
**Professional category**				
Nursing	131 (49.1%)	104 (47.9%)	0.875	484
Medical	136 (50.9%)	113 (52.1%)		
**Emotional exhaustion**				
Low (< 19)	154 (57.7%)	45 (20.7%)	<0.001	484
Medium (19–26)	51 (19.1%)	40 (18.4%)		
High (>26)	62 (23.2%)	132 (60.8%)		
**Depersonalization**				
Low (< 6)	170 (63.7%)	71 (32.7%)	<0.001	484
Medium (6–9)	64 (24.0%)	54 (24.9%)		
High (>9)	33 (12.4%)	92 (42.4%)		
**Professional accomplishment**				
Low (>39)	24 (8.99%)	58 (26.7%)	<0.001	484
Medium (34–39)	101 (37.8%)	93 (42.9%)		
High (<34)	142 (53.2%)	66 (30.4%)		

There are significant differences (*p* < 0.001) in the three categories of burnout. The high scores for emotional exhaustion and depersonalization increased, rising between 2014 and 2021 from 23.2 to 60.8% and from 12.4 to 42.4%, respectively. However, there is also a significant increase in high personal accomplishment, rising from 9% in 2014 to 26.7% in 2021.

### Emotional exhaustion

Emotional exhaustion is greatest among professionals aged 31–40 years working in rural areas. However, in urban areas, emotional exhaustion is worse among the 41–50 age group, and even more accentuated among professionals over 50 years of age.

**Figure 1:**
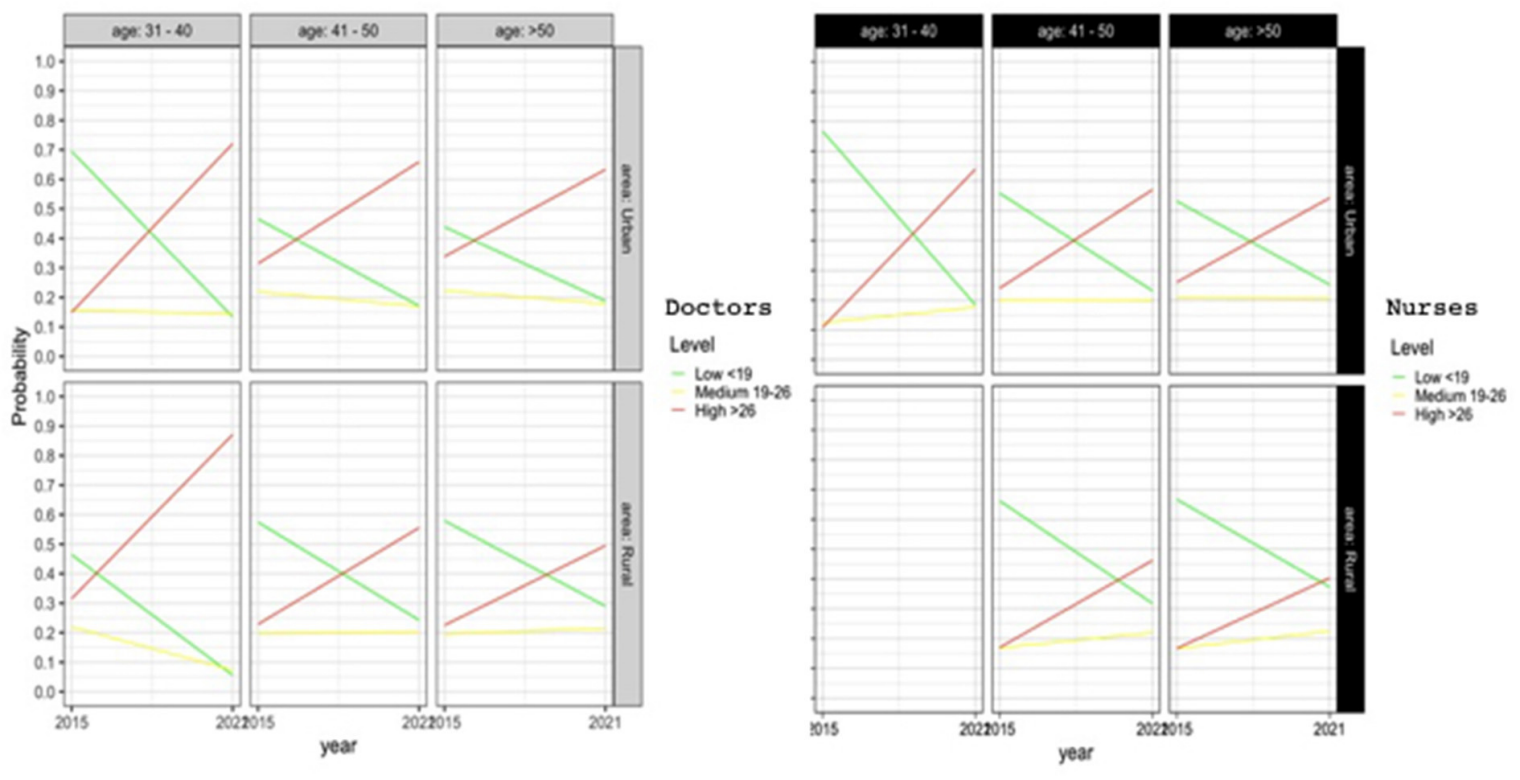
Logistic regression model for emotional exhaustion.

Physicians show higher scores of emotional exhaustion compared to nursing professionals. However, there are no significant differences regarding gender. Figure 1 shows how the number of professionals who obtain a high score for emotional exhaustion has risen at all ages, both in urban and in rural areas.

### Depersonalization

**Figure 2:**
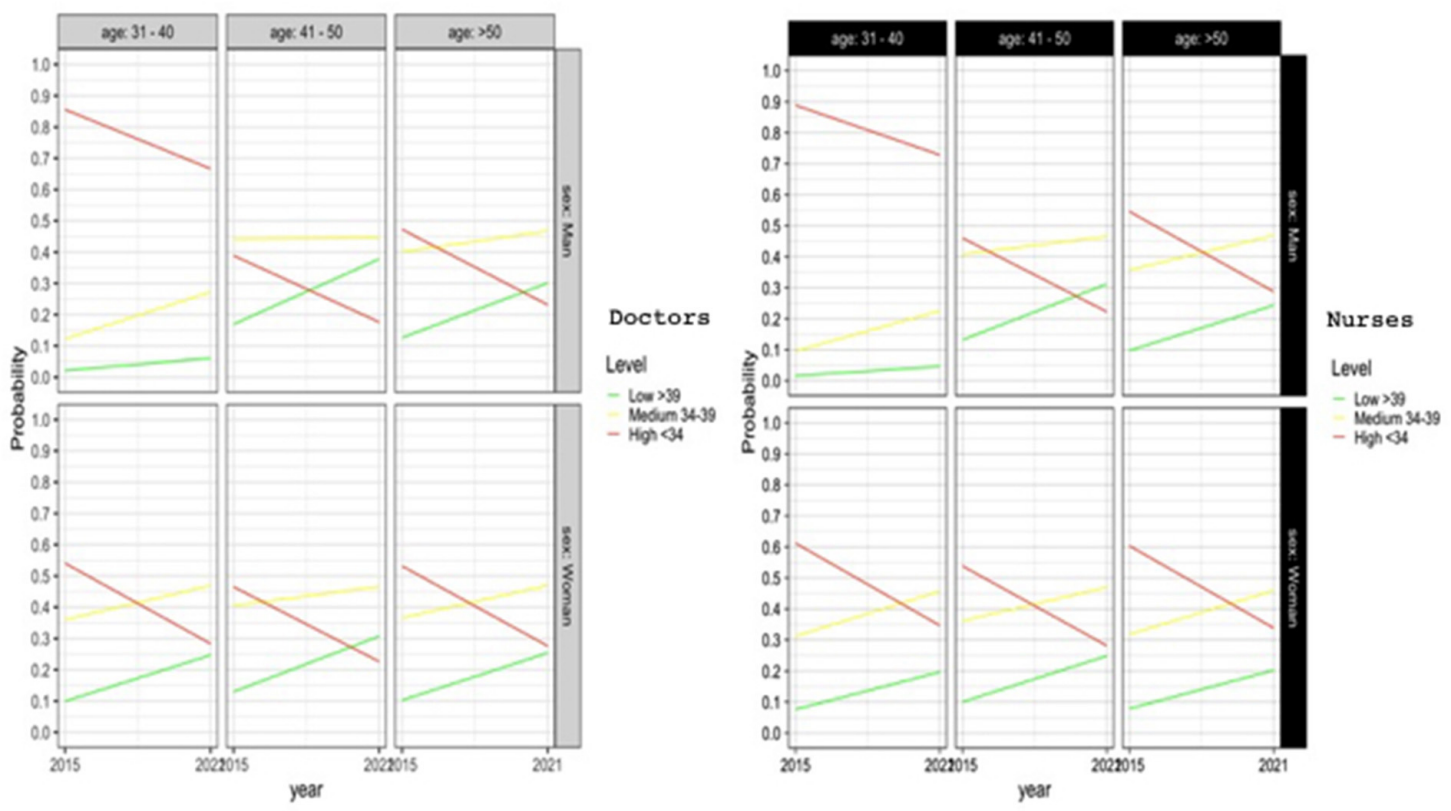
Logistic regression model for depersonalization.

Depersonalization is marked mainly by place of work. Depersonalization is worse in urban areas (*p* < 0.05). In rural areas, depersonalization has increased far less compared to 2014, as shown in Figure 2.

Male nurses display greater depersonalization than their female counterparts (*p* < 0.05). However, the differences are not significant in the case of physicians.

We have not detected any significant differences with regard to age groups (Figure 2).

### Professional accomplishment

Young women (31–40 years) show significantly greater professional accomplishment (*p* < 0.05). In the other age groups there are no significant differences. As for men, it is young males (31–40 years old) who present the least professional accomplishment (*p* < 0.05).

**Figure 3:**
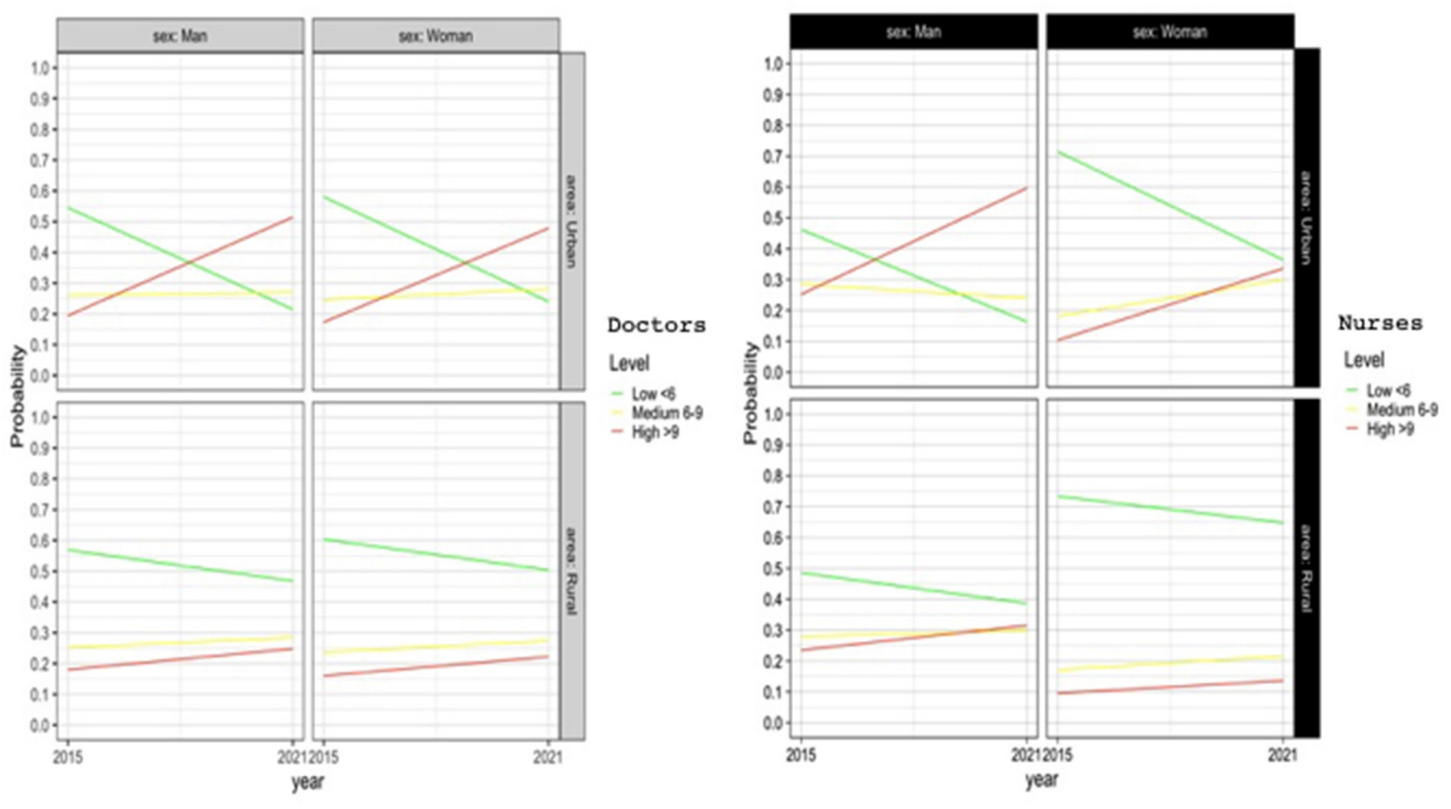
Logistic regression model for professional accomplishment.

However, professional accomplishment has improved significantly for both men and for women, as shown in Figure 3. More professionals obtain a higher score for professional achievement in 2021 compared to 2014. This improvement observed in 2021 can be considered similar according to gender, age, work environment or professional category. There are no statistically significant differences in professional accomplishment between work environment and category.

## Discussion

### Main findings

The study revealed significant differences in the three categories of burnout among primary care professionals between the 2014 and 2021, in mid-pandemic (*p* < 0.001). The scores for emotional exhaustion and depersonalization were very high during the pandemic year (2021) and increased compared to 2014 (4), indicating a significant impact of the pandemic on professional exhaustion. However, a significant increase in personal accomplishment was also observed, suggesting an adequate vocational response and the resilience of professionals during the pandemic year.

### Strengths and limitations

Our study does have some limitations. The sample of participating professionals, despite the study variables being the same, corresponds to professionals in professional practice with seven years of difference and with relatively different professional environments. Moreover, in 2021 the response rate was <50%. During the pandemic period, some respondents may have been influenced by the levels of transmission of SARS-CoV-2 at community level and the healthcare pressure in their health center or work environment. In addition, to preserve their anonymity, the age of the participants was not collected individually. Finally, we have not been able to obtain the data for the global scales of 2014, but we believe that the differences between 2014 and 2021 are large enough, and would probably go in the same direction. However, one strength of our study is that we have two samples to be able to compare this evolution. This allows us to know the professionals' status in 2014 and their status in 2021.

### Interpretation of the study results

These burnout levels among primary care professionals in the population studied in 2021 are very high, with high MBI scores in almost 70% of cases. The results are higher than those found in other studies carried out previously (20) and also those described in 2014 concerning primary healthcare workers in Lleida, which presented comparable sociodemographic characteristics to the population studied in 2021 (4). Professional burnout found for 2021 is higher than expected, coinciding with the period of the SARS CoV-2 coronavirus pandemic.

The high incidence of burnout among primary care physicians and nursing staff in the period of the pandemic highlights the high emotional impact it has caused among workers and is possibly also a reflection of previous shortcomings in our health system regarding which there is a need for mandatory changes, such as reducing bureaucratic tasks, promoting professionals' implication in management and increasing salaries for healthcare professionals. Moreover, burnout is a result of many factors. Of course excessive workloads and inherent stresses of healthcare professionals are important, but so are social structure, degree of social reserve or coping mechanisms, especially among students.

Emotional exhaustion is one of the three axes of burnout that is most notably suffered by health professionals and in various studies (21, 22). Factors such as workload have been seen to be associated with higher levels of emotional exhaustion (23).

This correlates with the data found in our study in which emotional exhaustion has also been observed to be more affected by age and professional group.

Several studies show statistically significant differences for gender and age. Being of female gender and of a younger age were seen as risk factors for developing burnout (5, 8). In our comparison there are no statistically significant differences in terms of gender. However, higher levels of emotional exhaustion have been observed in young people aged 31–40 years working in rural areas, which could be explained by a relatively more isolated professional practice with less team relations.

Meanwhile, in the group aged over 41 years, emotional exhaustion worsens in urban areas, which could be explained by professional practices in environments with greater pressure on healthcare services by elderly persons that may result in less responsiveness to excessive labor demands.

During the SARS-CoV2 pandemic, statistically significant differences have been detected between physicians and nursing staff, the former being the most affected with twice the risk of suffering emotional exhaustion. These data related to professional category coincide with those detected in the study of 2014 (4) and could be linked to the uncertainty in decision-making that characterizes the practice of family medicine.

Statistically significant differences have been found for depersonalization in relation to the workplace variable, which is more frequent in urban areas compared to rural ones where depersonalization has increased to a lesser extent compared to the data for 2014. Professional practice in urban environments may be related to a highly protocolized exercise involving less professional initiative which has been suggested as a possible risk factor for high levels of depersonalization.

In 2021, high rates of emotional exhaustion were found (58.2%) but, interestingly, a moderate-high level was also observed (45.5 + 25.8%; the sum is 70%) in terms of personal accomplishment. This has already been described in previous studies, such as the systematic review by García-Iglesias et al. (16), which reveals that there are high levels of burnout among health professionals but, paradoxically, also high levels of job engagement, or personal accomplishment.

This may be because working under extreme circumstances like the Covid pandemic got the best out of the healthcare workers, who perhaps experienced a genuine vocational calling. Being able to help a large number of people and feeling useful by working intensely could be one of the reasons that made the levels of personal accomplishment also high. All of the above, despite the fact that working conditions could be greatly improved to prevent the other two components of burnout from being affected so negatively.

This is also reflected in studies during the pandemic like the one by Giménez-Espert et al. (20), in which they highlighted high levels of nurses' labor commitment and levels of satisfaction that may be due to factors like resilience and personal satisfaction by being aware of the importance of their work to society.

Therefore, one might imagine that personal accomplishment can function as an independent entity within the components of burnout, moving away from emotional exhaustion and depersonalization in certain challenging work situations.

Even so, as established by Wildgruber et al. (21), personal accomplishment decreases parallel to the increase in cumulative stress related to the pandemic. Therefore, this should be taken into account to prevent the degree of personal accomplishment from deteriorating and exacerbating burnout.

The results reflected in the present study suggest that the work-related demands on health professionals resulting from the pandemic situation have often been excessive and most exacting. This may have caused some health professionals to feel overwhelmed and that they lacked personal resources to deal with certain situations. As reflected in the *Job Demands—Resources* (JD-R) model (18), which explains that health professionals in the performance of their duties are influenced by labor demands and the resources available to them at a personal, situational or organizational level, one might be led to believe that the pandemic situation has revealed a deterioration in working conditions that had remained hidden for some time in our health system. The crisis situation caused by the pandemic has acted as a mirror that has reflected the lack of resources at situational and organizational levels that already existed, but may not have been so clearly visible. This lack of resources at organizational level has also meant that the personal strategies of some professionals to withstand this situation have been greatly altered and this translates into such a massive increase in burnout reaching previously unrecorded levels.

### Leadership and resilience as an implication of the research

It is important to know the state of burnout to establish the needs of the system and to subsequently propose the interventions that require implementing in order to improve the working conditions of healthcare professionals. The new models of clinical leadership (24) that have emerged since the pandemic are challenged with reducing the stress levels of professionals and taking care of the professionals who have been on the front line during all waves of the pandemic. In addition, the provision of training to increase resilience and empathy might also help protect healthcare workers from burnout. This could perhaps be implemented at university medical and nursing faculties, given the high levels of burnout detected among medical and nursing students.

## Conclusion

In conclusion, the study confirms a very significant emotional impact of the SARS-CoV2 pandemic on health professionals, worsening burnout levels, specifically emotional exhaustion and depersonalization. However, it also shows that during the pandemic, personal accomplishment has been strengthened, possibly thanks to the resilience and the professional vocation of a large proportion of healthcare workers. This should be taken into consideration by those in charge of the relevant health organizations.

Therefore, it is important to evaluate the conditions and elements that promote professional accomplishment as well as to analyse the factors that can alter the resources that increase the level of burnout, in order to guarantee a quality health system that provides better care to patients and better employment and personal conditions of health professionals that allow them to carry out their work adequately. This means that our health system must undergo reforms to protect health professionals, especially in the light of the impact that the pandemic has had on them.

## Data availability statement

The raw data supporting the conclusions of this article will be made available by the authors upon reasonable request.

## Ethics statement

The studies involving human participants were reviewed and approved by IDIAP Jordi GOL. The patients/participants provided their written informed consent to participate in this study.

## Author contributions

AA created the data base. AF and EP lead the project. SG and SP realized the data analysis and collaborate in the draft of the manuscript. OY reviewed the manuscript and did data curation. All authors contributed to the article and approved the submitted version.

## References

[1] International Classification of Diseases (ICD 11). (2018). Available online at: https://icd.who.int/es/docs/Guia%20de%20Referencia%20(version%2014%20nov%202019).pdf (accessed December 8, 2022).

[2] Grau Martin A. Como Prevenir el Burnout: Diferentes Definiciones e Interpretaciones. (2007). Available online at: http://www.recercat.cat/handle/2072/94980 (accessed February 5, 2021).

[3] Navarro Gonzalez D Ayechu Diaz A Huarte Labiano I. Prevalencia del Síndrome de Burnout y Factores Asociados a Dicho Síndrome en los Profesionales Sanitarios de Atención Primaria. Medicina de Familia. Madrid: SEMERGEN. (2015). 10.1016/j.semerg.2014.03.008 24857630

[4] Yuguero Torres O. Estudio de la Empatia y Burnout de los Medicos y Enfermeras de Atencion Primaria de la Region Sanitaria de Lleida y su Relacion con las Variables Clinicas. (2015). Available online at: http://www.tdx.cat/handle/10803/307054 (accessed February 1, 2021).

[5] Karuna C Palmer V Scott A Gunn J. Prevalence of burnout among GPs: a systematic review and metaanalysis. Br J Gen Pract. (2022) 72:e316–24. 10.3399/BJGP.2021.0441 34990391 PMC8869191

[6] Nituica C Bota OA Blebea J. Specialty differences in resident resilience and burnout - a national survey. Am J Surg. (2021) 222:319–28. 10.1016/j.amjsurg.2020.12.039 33431168

[7] Dosil Santamaria M Ozamiz-Etxebarria N Redondo Rodriguez I Jaureguizar Alboniga-Mayor J Picaza Gorrotxategi M. Psychological impact of COVID-19 on a sample of Spanish health professionals. Rev Psiquiatr Salud Ment. (2021) 14:106–12. 10.1016/j.rpsmen.2020.05.002 32622882 PMC7264016

[8] Erquicia J Valls L Barja A Gil S Miquel J Leal-Blanquet J. Emotional impact of the Covid-19 pandemic on healthcare workers in one of the most important infection outbreaks in Europe. Med Clínica. (2020) 155:434–40. 10.1016/j.medcle.2020.07.010 33163628 PMC7604105

[9] Alonso J Vilagut G Mortier P Ferrer M Alayo I Aragón-Peña A. Mental health impact of the first wave of COVID-19 pandemic on Spanish healthcare workers: a large cross-sectional survey. Rev Psiquiatr Salud Ment. (2021) 4:90–105. 10.1016/j.rpsm.2020.12.001 33309957 PMC7726524

[10] Xiao Y Chen J. Job burnout facing Chinese general practitioners in the context of COVID-19. Int J Qual Health Care. (2022) 34:mzac023. 10.1093/intqhc/mzac023 35349679 PMC8992304

[11] Moreno-Jiménez B Carvajal RR Escobar RE. La evaluación del Burnout profesional. Factorializa-cion del MBI-GS Un análisis preliminar. Ansiedad Estrés. (2001) 7:69–78.

[12] De las Cuevas C. El Desgaste Profesional en Atención Primaria: Presencia y Distribución del Síndrome de “Burnout”, 1st ed. Madrid: Lab Servier (1997).

[13] Yuguero O Marsal JR Esquerda M Soler-González J. Occupational burnout and empathy influence blood pressure control in primary care physicians. BMC Fam Pract. (2017) 18:63. 10.1186/s12875-017-0634-0 28499346 PMC5429573

[14] Yuguero O Forné C Esquerda M Pifarré J Abadías MJ Viñas J. Empathy and burnout of emergency professionals of a health region: a cross-sectional study. Medicine. (2017) 96:e8030. 10.1097/MD.0000000000008030 28906390 PMC5604659

[15] Yuguero Torres O Esquerda Aresté M Marsal Mora JR Soler-González J. Association between sick leave prescribing practices and physician burnout and empathy. PLoS ONE. (2015) 10:e0133379. 10.1371/journal.pone.0133379 26196687 PMC4510532

[16] García-Iglesias JJ Gómez-Salgado J Fagundo-Rivera J Romero-Martín M Ortega-Moreno M Navarro-Abal Y. Factores predictores de los niveles de burnout y work engagement en médicos y enfermeras: una revisión sistemática [Predictive factors for burnout and work engagement levels among doctors and nurses: a systematic review.]. Rev Esp Salud Publica. (2021) 95:e202104046. 33818557

[17] Adriaenssens J De Gucht V Maes S. Association of goal orientation with work engagement and burnout in emergency nurses. J Occup Health. (2015) 57:151–60 10.1539/joh.14-0069-OA 25735623

[18] Montgomery A Spânu F Băban A Panagopoulou E. Job demands, burnout, and engagement among nurses: a multi- level analysis of ORCAB data investigating the moderating effect of teamwork. Burn Res. (2015) 2:71–9. 10.1016/j.burn.2015.06.001 26877971 PMC4710673

[19] R Core Team. R: A Language Environment for Statistical Computing. Vienna, Austria: R Foundation for Statistical Computing (2021). Available online at: https://www.R-project.org/ (accessed October 9, 2022).

[20] Solms L Van Vianen AEM Theeboom T Koen J De Pagter APJ De Hoog M. Keep the fire burning: a survey study on the role of personal resources for work engagement and burnout in medical residents and specialists in the Netherlands. BMJ Open. (2019) 9:e031053. 10.1136/bmjopen-2019-031053 31694848 PMC6858141

[21] Wildgruber D Frey J Seer M Pinther K Koob C Reuschenbach B. Work engagement and stress experience of health professionals in times of the corona pandemic: a cross-sectional study. Pflege. (2020) 33:299–307. 10.1024/1012-5302/a000759 32996863

[22] Giménez-Espert MDC Prado-Gascó V Soto-Rubio A. Psychosocial risks, work engagement, and job satisfaction of nurses during COVID-19 pandemic. Front Public Health. (2020) 8:566896. 10.3389/fpubh.2020.566896 33330313 PMC7716584

[23] Demerouti E Nachreiner F Bakker AB Schaufeli WB. The job demands-resources model of burnout. J Appl Psychol. (2001) 86:499–512. 10.1037/0021-9010.86.3.499 11419809

[24] Yuguero O Inzitari M Tolchinsky G. Clinical leaders, the first step for emotionally intelligent leadership. BMJ. (2022) 6:219–21. 10.1136/leader-2020-000423 36170485

